# Leaching Stability and Redox Activity of Copper-MFI Zeolites Prepared by Solid-State Transformations: Comparison with Ion-Exchanged and Impregnated Samples

**DOI:** 10.3390/ma16020671

**Published:** 2023-01-10

**Authors:** Svetlana A. Yashnik, Tatjana A. Surovtsova, Anton V. Salnikov, Valentin N. Parmon

**Affiliations:** Boreskov Institute of Catalysis SB RAN, Prospekt Akademika Lavrentieva, 5, 630090 Novosibirsk, Russia

**Keywords:** Cu-ZSM-5, copper-containing silicalite, preparation route, oxide cluster of Cu^2+^ cations, CuO nanoparticle

## Abstract

The catalyst preparation route is well known to affect the copper loading and its electronic state, which influence the properties of the resulting catalyst. Electronic states of copper ions in copper-containing silicalites with the MFI-framework topology obtained by a solid-state transformation S (SST) were studied with using EPR, UV-Vis DR, XRD, H_2_-TPR and chemical differentiating dissolution. They were compared with Cu-ZSM-5 and Cu-MFI (silicalite) prepared via the ion-exchange and incipient wetness impregnation. SST route was shown to provide the formation of MFI structure and favor clustering of Cu-ions near surface and subsurface of zeolite crystals. The square-planar oxide clusters of Cu^2+^-ions and the finely dispersed CuO nanoparticles with the size down to 20 nm were revealed in Cu-MFI-SST samples with low (0.5–1.0 wt.%) and high (16 wt.%) Cu-content. The CuO nanoparticles were characterized by energy band gap 1–1.16 eV. The CuO-like clusters were characterized by ligand-to-metal charge transfer band (CTB L → M) at 32,000 cm^−1^ and contain EPR-visible surface Cu^2+^-ions. The low Cu-loaded SST-samples had poor redox properties and activity towards different solvents due to decoration of copper-species by silica; whereas CuO nanoparticles were easily removed from the catalyst by HCl. In the ion-exchanged samples over MFI-silicalite and ZSM-5, Cu^2+^-ions were mainly CuO-like clusters and isolated Cu^2+^ ions inside MFI channels. Their redox properties and tendency to dissolve in acidic solutions differed from the behavior of SST-series samples.

## 1. Introduction

The copper-substituted MFI zeolites have attracted strong attention due to their ability to catalyze the decomposition of NO and N_2_O [1,2] as well as selectively reduce NOx to N_2_ with hydrocarbons [3,4] and ammonia [5,6] in the oxygen presence. Today, copper-containing MFI is intensively considered as a promising catalyst for catalytic wet peroxide oxidation (CWPO) of organic contaminants, such as phenol and textile dyes [7,8,9,10,11,12]. Cu-ZSM-5 zeolites were shown to be more efficient than Fe-containing ones in CWPO of formic acid, phenol and Rhodamine 6G [7,8,10,13]. The former can provide the complete destruction of phenol and high mineralization of total organic compounds without leaching of a considerable quantity of copper to the solution [5]. Cu-modified MFIs catalyze the oxidation of methane with hydrogen peroxide [14,15,16] or oxygen [17,18,19] to methanol and other valuable products, although these catalysts have not yet found practical applications in this area.

The applicability of the Cu-ZSM-5 catalyst for the CWPO reactions can be limited by the low stability of catalytically active ions to leaching from the zeolite matrix into the liquid. The leaching of copper cations from the zeolite causes deterioration in its catalytic characteristics as well as lifetime. In this connection, the detailed information on the resistance of Cu-species stabilized inside zeolite channels and over their surface to leaching in water and acidic solution is relevant.

The catalytic, redox and physicochemical properties of the Cu-containing zeolites are well known to significantly depend on the catalyst preparation mode, which controls the copper electronic states and their loadings. The resistance of the various copper species to leaching in aqueous solutions is inferred also function of preparation mode. However, a comparison of single scattered data shows that the “preparation mode–structure–leaching” relationships often completely contradict each other. For example, the stability of the Cu/ZSM-5 catalyst prepared by direct hydrothermal synthesis was better than stability of the ion-exchanged catalyst because the leaching of active ingredient was lower [9]. On the other hand, we observed 3–5 times less removal of copper from the ion-exchanged Cu-ZSM-5 catalysts during CWPO of formic acid compared with the Cu-MFI zeolites obtained by solid-state transformation [10]. For the ion-exchanged catalysts, there has been a tendency to a 2–15-fold increase in the amount of copper ions leaching with increasing a copper loading and Si/Al ration of the zeolite [10]. The reason for the different resistance to leaching from Cu-zeolite can be related to the various copper electronic state formed during the synthesis.

Post-synthetic modification of the zeolites through an ion-exchange in solution (‘wet’) or in the solid-state phase (‘dry’) is the widely used method for the preparation of copper-containing zeolites. The ion-exchange of the ZSM-5 zeolites in H-, Na- or NH_4_-forms with a copper (II) salt solution (usually acetate, nitrate or chloride [1,20,21,22]) or with solid-state copper compound (for example, chloride or oxide [23,24,25,26,27]) yields Cu-ZSM-5 with the copper loading equivalent to the exchange level up to 100% (or Cu/Al ≤ 0.5). Such Cu-ZSM-5 catalysts maintain mainly copper as isolated Cu^2+^-ions, which compensated a charge of zeolite lattice and located in the cation-exchange sites inside the zeolite channels [20,22,28,29,30]. In contrast to ZSM-5, silicalite-1, which is silica with MFI structure [31], possesses a greatly diminished ability to the ion-exchange with aqueous copper solution [31] due to the lack of aluminum ions in its lattice. Therefore, copper ions in silicalite-1 are usually loaded by the grafting from water-ammonia solutions [32] or by the ion-exchange with copper acetate solution at 50 °C [33,34]. Up to 85% of the grafted copper ions are stabilized presumably on the silanol groups of the external silicalite surface [32] and supermicropores [34], transforming to the low-nuclearity CuO-like clusters at thermal treatment [33,34]. The other copper ions are in positions similar to those in CuZSM-5 [32].

Repetition of the ion-exchange procedure [1] or the application of an ammonia-copper salt solution can result in Cu-ZSM-5 zeolite with higher copper loading [20,21]. In so-called over-exchanged Cu-zeolites, the copper exchange level exceed 100% and even can reach 200–400% (Cu/Al = 1–2) [1,20]. For them, the predominant copper state was found to be copper structures with extra-lattice oxygen, for example dimers ([Cu-O-Cu]^2+^), oxo-complexes ([Cu-(O)_2_-Cu]^2+^) and chain-like structures inside the zeolite channels as well as highly dispersed copper-oxide clusters (Cu_x_O_y_) on the zeolite surface [20,25,35,36].

Another method of copper loading is a hydrothermal synthesis of the zeolite in the presence of copper salts [9,37,38,39]. It was directed on isomorphous substitution of the Si^4+^ cations of the MFI lattice on Cu^2+^ cations [37]; however, Cu^2+^ cations were shown [38] to do not enter into the zeolite framework and have an octahedral coordination. A relatively new method for the preparation of metal-containing MFI with high metal (Al, Co, Cu, et al.) content is solid-state transformation (SST) [40,41,42,43]. It resembles the hydrothermal crystallization of the Cu-modified silica-alumina hydrogel. The SST synthesis of the MFI framework includes the addition of copper, cobalt or aluminum nitrate directly to a silicon source (usually kanemite, which is a hydrated layered sodium silicate NaHSi_2_O_5_·3H_2_O) and aqueous solution of tetrapropylammonium hydroxide (TPAOH), followed by heating the metal-kanemite-TPAOH powder in a closed glass reactor at 130 °C [41,42]. Similar to the hydrothermal crystallization, the isomorphous substitution of Si^4+^ cation resulted in the tetrahedral coordination by Cu^2+^ ions was absent [38]; meanwhile, small MFI-like crystals with the highly dispersed copper inside bulk zeolite were found to form during the SST synthesis [42]. However, the actual state of copper in these Cu-SST catalysts was not discussed earlier.

The incipient wetness impregnation is the most practically feasible method for the preparation of Cu-ZSM-5 with high copper loading (5 wt.% and higher, Cu/Al > 2). Important is that large copper-oxide clusters as well as finely dispersed copper oxide particles formed via this procedure are located on the external surface of zeolite crystallites [10,22,44].

By analogy with Fe-containing silicalite, it is assumed [9,43,45] that copper ions embedded in the zeolite framework by hydrothermal synthesis or SST are leached more difficultly from Cu-MFI zeolite in aqueous solutions. However, as it was shown in our work [10], the ion-exchanged Cu-ZSM-5 zeolites were more stable to leaching than the Cu-MFI prepared by SST. In the present work, we have attempted to find out the reason for this. To this purpose, the relationships between the structure and the leaching of copper in Cu-containing zeolites prepared by SST and the ‘wet’ ion-exchange with silicalite-1 are examined in detail by temperature-programmed reduction by hydrogen (H_2_-TPR), electron paramagnetic resonance (EPR) and Ultraviolet-visible Diffuse Reflectance (UV-Vis DR) spectroscopy as well as chemical differentiating dissolution (CDD). The obtained data are compared to those for the ion-exchanged and the impregnated Cu-ZSM-5 with the similar copper loading; copper states in them are widely discussed in earlier works [7,20,21,25,28,29,30,35,36].

## 2. Materials and Methods

### 2.1. Materials

Copper(II) nitrate 3-hydrate (Cu(NO_3_)_2_·3H_2_O, >99% purity), copper(II) acetate monohydrate (Cu(CH_3_COO)_2_·H_2_O, ≥98% purity), CuO (very-high-purity), concentrated nitric acid (75% HNO_3_, high-purity), ammonia solution (25% NH_4_OH, very-high-purity), hydrochloric acid (35% HCl, high-purity) and hydrofluoric acid (45% HF, high-purity) were purchased from Reachem (Moscow, Russia). Tetrapropylammonium hydroxide (TPAOH) was purchased from Aldrich Chemical Co., Inc. (Merck, Darmstadt, Germany)

### 2.2. Catalyst Preparation

The first series of Cu-MFI samples was prepared by solid-state transformation. An amount of 7 mL of an aqueous solution of TPAOH (26 wt.%) was added to a kanemite (1 g, as powder) at continuous stirring. The mixture was stirred until a homogeneous suspension was obtained. Then, a 3 mL of solution containing 19–615 mg Cu(NO_3_)_2_·3H_2_O was added, and the suspension was actively mixed at 70 °C for 3 h. The suspension was cooled to room temperature, and its pH was lowered from 13.0 to 8.5 by nitric acid. The formed solid deposit was filtered, washed with distilled water, and dried at 25 °C for 18 h. The resulting product was placed in a 5-mL glass ampoule, sealed, and heated at 130 °C for 72 h. After the SST, the samples were heated to 500 °C with rate 2°/min and calcined at 500 °C for 20 h in flowing air.

The second series of Cu-MFI samples was prepared by the ion-exchange of silicalite-1 (Si/Al < 150, Si/Fe < 1100) with water-ammonia solution of copper acetate at room temperature. Solutions had pH 10.4 and copper concentrations equal to 2.0 and 6 mg Cu/mL. The slurry concentration (the ratio of the solution volume to the zeolite mass, S/Z) was 10. The duration of ion exchange was 48 h. Then, the zeolite samples were filtered and washed with distilled water. The samples were dried at 110 °C for 8 h and calcined in air at 500 °C for 5 h (heating rate 2 °/min).

The third series of Cu-ZSM-5 samples was prepared by the ion-exchange of parent H-ZSM-5 (Si/Al = 30) with aqueous copper acetate solutions, which had pH close to 5.75 and copper concentrations equal to 0.5, 2.0, and 8 mg Cu/mL, at room temperature according to [20]. The slurry concentration was 10. After 48 h of ion exchange, the zeolite samples were filtered and washed with distilled water. The sample washing was stopped when a blue coloring of wash water at ammonia addition became absent. All the samples were dried at 110 °C for 8 h, heated with 2 °/min and calcined in air at 500 °C for 5 h.

Cu/H-ZSM-5 samples were also prepared by the incipient wetness impregnation of the H-ZSM-5 (Si/Al = 30) powder with an aqueous copper acetate solution with the concentration equivalent to 2 and 16 wt.% of the copper loading. Then, the samples were dried at 110 °C for 8 h and calcined in air at 500 °C for 5 h (heating rate was 2 °/min).

The copper content in the Cu-zeolite samples was determined by the atomic emission spectrometry with the inductively coupled plasma method (ICP-AES Instrument, Perkin-Elmer Optima DV-3300, Perkin Elmer Inc., Waltham, MA, USA). The copper content in the samples was near 0.5; 1.0; 2.0; and 16 wt.%.

Below, the samples are denoted as n%Cu-Z-X, where n% is copper content (in wt.%), Z is zeolite topology: MFI—for silicalite and ZSM-5—for alumosilicalite; X is the route of Cu introduction into zeolite: SST—solid-state transformation, IEX—ion-exchange, and IMP—incipient wetness impregnation. The chemical compositions of Cu-zeolite are presented in Table 1.

### 2.3. Catalyst Characterization

X-ray diffraction analysis (XRD) of the samples was carried out using an ARL X’TRA diffractometer (Thermofisher Scientific Inc., Ecublens, Switzerland) with monochromatic CuK_α_ radiation (λ = 1.54178 Å) by scanning with 0.05° step in the range of Bragg angles (2θ) 5–50° with the 3-s accumulation at each point.

EPR spectra of the water-saturated samples were recorded using a Bruker EMX Micro 6/1 spectrometer (Bruker, Karlsruhe, Germany) with microwave region λ = 3 cm, high-frequency modulation of magnetic field—100 kHz and magnetic field up to 5000 G at 77 and 298 K in a quartz glass ampoule with an internal diameter of 3 mm. The parameters of the EPR spectra were determined by comparison with the spectrum of a DPPH standard (diphenylpicrylhydrazyl, g = 2.0037 ± 0.0002). The amount of spins present in the Cu-zeolites was determined by the analysis of the second integrals of the measured first derivative EPR spectra. The accuracy of such assessment is ~30%. The concentration of EPR-visible Cu(II) ions (spin/g) was calculated taking into account the weight of the Cu-zeolite.

UV-Vis DR spectra were obtained at room temperature using a Shimadzu UV-2501 PC spectrophotometer (Shimadzu, Kyoto, Japan) equipped with a diffuse reflectance accessory (ISR-240 A). The spectra were recorded against a BaSO_4_ reflectance standard in the range of 900–190 nm (11,000–54,000 cm^−1^). Below, the UV-Vis DR spectra are shown in the Kubelka-Munk units: F(R_∞_) vs. wavenumber.

H_2_-TPR experiments were conducted using a flow-reactor connected with a thermal conductivity detector. A Cu-containing MFI sample (100 mg, fraction 0.25–0.50 mm) was placed in the reactor, pretreated in the oxygen flow (30 cm^3^/min) at 400 °C for 0.5 h and cooled to room temperature. Then, a hydrogen-argon mixture (10 vol.% H_2_) was passed through the Cu-MFI sample as the reducing agent with feed rate 30 cm^3^/min. The temperature range and heating rate were 25–630 °C and 10 °C/min, respectively. Water formed during the reduction in the copper-oxygen species was frozen out in a trap at −70 °C. The H_2_ consumption was calibrated against the reduction in CuO at similar conditions, assuming the complete one-step CuO reduction to zero-valent copper.

Chemical differentiating dissolution (CDD) of the Cu-MFI samples was performed in a flow reactor at dynamic conditions with the stepwise variation of the solvent nature [46]. Water with a temperature of 22 °C was used as the first solvent, the 1.2 M hydrochloric acid solution heated to 40 °C was used as the second one, 3 M hydrochloric acid solution heated to 65 °C was used as the third one, and the hydrofluoric acid solution (1:5) with a temperature of 80 °C was applied as the fourth one. The solvent flow rate was 3.6 cm^3^/min.

## 3. Results and Discussion

### 3.1. Leaching Stability

The Cu-zeolites are well known to be characterized by the leaching effect of copper ions in aqueous solutions. The leaching effect is enhanced by lowering the pH of aqueous solutions. As our experience shows [8,10,11,12], the leaching effect is more destructive for Cu-zeolite, when inorganic acids are formed during CWPO of the organic ecotoxicant, for example, the hydrochloric acid or nitric acid at degradation of Rhodamine 6G [8,13,47]. For example, the copper leaching from 1%Cu-ZSM-5-IEX was as high as 41% after CWPO of Rhodamine 6G [13], and near 10% after CWPO of formic acid [10] and phenol [11].

**Figure 1:**
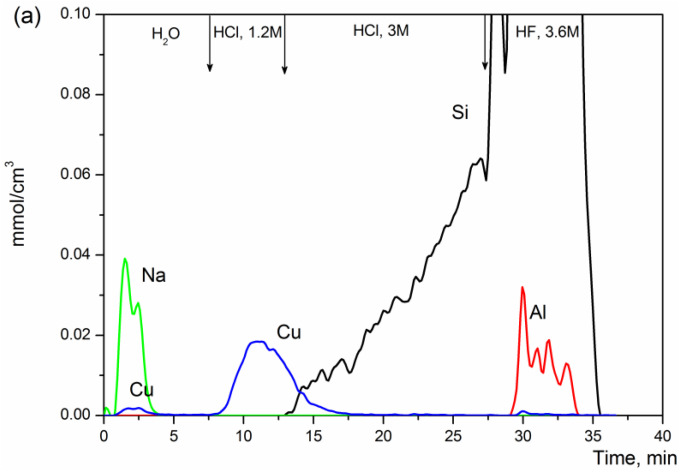

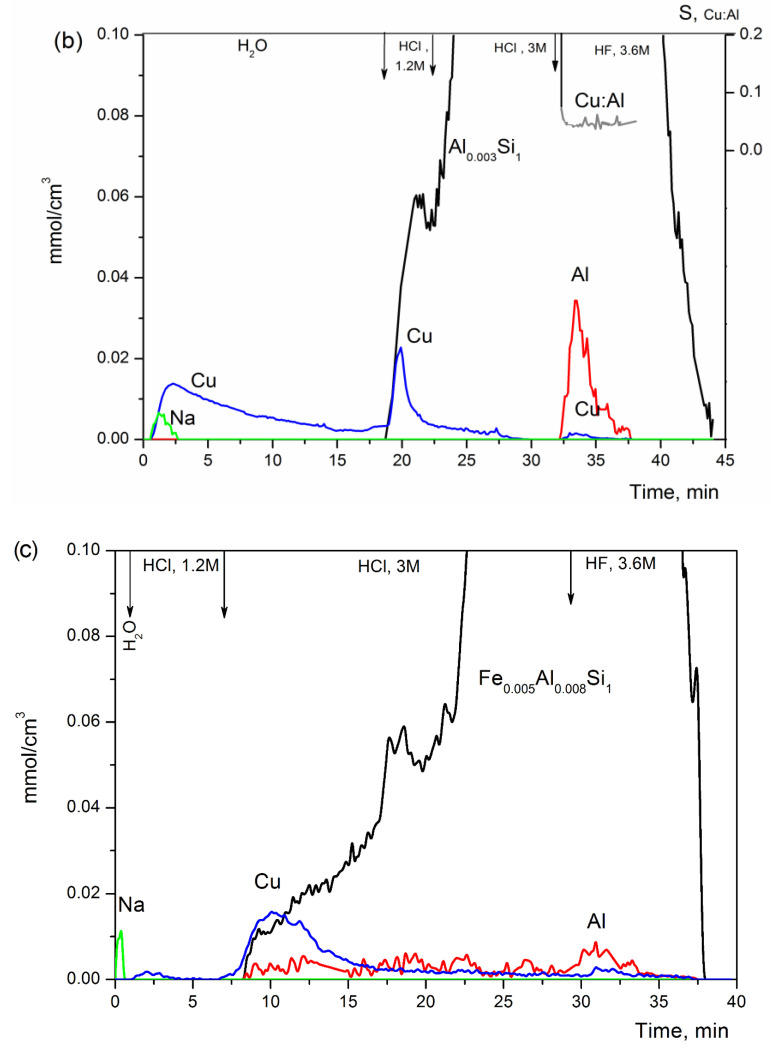
Differential dissolution curves of Cu-containing MFI prepared via solid-state transformation ((**a**): 2%Cu-MFI-SST) and ion-exchange ((**b**): 2%Cu-MFI-IEX, (**c**): 2%Cu-ZSM-5-IEX) modes.

Here the leaching effects of Cu-zeolites were studied by the chemical differentiating dissolution, using dynamic conditions with the stepwise change of the solution pH due to variation of solvent from water to HCl and HF solutions. Figure 1 illustrates the kinetic curves of the dissolution of the Cu-containing zeolites prepared by different mode. Table 2 summarizes a hypothesized structural phase composition and its quantitative content in Cu-zeolites.

Two forms of copper located on the surface and in the subsurface layers were discovered in the 2%Cu-MFI-SST sample by the differentiating dissolution technique (Figure 1a). The first form (7% of total Cu) is dissolved in water simultaneously with sodium present in the sample. The second form (91% of total Cu) starts dissolving in the 1.2 M HCl solution and completely dissolves in the 3M HCl solution. Copper was practically absent in the bulk of the silicalite matrix that slowly dissolved in 3M HCl and quickly dissolved in hydrofluoric acid (Figure 1a, curve Si). Its concentration in this phase did not exceed 2% of total copper. Note, the sample 2%Cu-MFI-SST contained an impurity of aluminum, and 0.14 wt.% was determined by ICP-AES (Table 1). Aluminum apparently entered a small amount into the silicalite framework as well as formed a separate phase. The molar stoichiogram calculated from kinetic curves of Si and Al dissolution was characterized by only a small section with a constant value of Al/Si ratio. Its value was about 0.0008 ± 0.0002. On the other parts of the stoichiogram, the Al/Si ratio fluctuated strongly, indicating the presence of the individually dissolved Al, probably as (AlO)_x_-like clusters.

Similar tendencies were observed for the Cu-MFI-SST sample with the higher copper concentration (16 wt.%). Most copper (98% of total Cu) was still present on the surface and in the subsurface layers of this sample, dissolving in water and then in 1.2 M solution of HCl (Table 2). Only a tiny fraction of copper (2%) was dissolved together with silicalite. On the other hand, the decrease in the copper concentration in Cu-MFI-SST results in a redistribution of copper forms. In particular, the fraction of copper dissolved together with silicalite in 3M HF increased from 2% to 15–30% of total copper (Table 2). However, the dissolution kinetics was still different from that observed for the ion-exchanged samples.

The 2%Cu-MFI-IEX sample prepared by post modification of silicalite was characterized by three peaks of copper dissolution in CDD experiment (Figure 1b). As in the case of the 2%Cu-MFI-SST sample, there was the copper species (65% of total Cu, Table 2) dissolving in water together with sodium present in the sample. Although, here the first copper type is dissolved by water more slowly compared with sample 2%Cu-MFI-SST. The second type (33% of total Cu) was extracted in 1.2 M solution of HCl simultaneously with 9% of silicalite. The third form of copper (2%) and 91% of silicalite, whose phase composition had the fragmental formula Al_0.003_Si_1_, were dissolved by 3.6 M HF solution. Copper was evenly distributed in the bulk of silicalite relative to aluminum (Cu_0.04_Al_1_). The sample also contained the individually dissolved Al, probably (AlO)_x_-like clusters.

Two forms of copper were also observed in the 2%Cu-ZSM-5-IEX sample prepared by the ion exchange (Figure 1c). There is absent copper dissolved in water. This sample dissolved slower and in more acidic solvents compared with the behavior of the Cu-MFI-SST and Cu-MFI-IEX samples. The first type (2.7% of total copper) is extracted by the 1.2 M HCl solution. The second form (97.3% of total Cu) extracts together with silicon, aluminum and iron in the 3 M HCl and HF until complete dissolution of the sample. The molar stoichiograms of Fe/Si and Al/Si had constant values, which were about 0.005 ± 0.001 and 0.008 ± 0.002, respectively. If the first copper-containing form is located on the surface, the second one is probably located in the subsurface layers and in the bulk of the zeolite.

Thus, the results obtained by the differential dissolution technique led us to a conclusion that most copper in the SST-series samples with copper loading 2 wt.% or more and in the ion-exchanged MFI-series is located on the surface or in the subsurface layers, whereas in the ion-exchanged Cu-ZMS-5 samples, copper is localized predominantly in the bulk of the sample. Further, we are trying to relate a copper leaching effect to its localization and electronic state in Cu-zeolites.

### 3.2. XRD Study of Cu-Containing Silicalites and Alumosilicalites with MFI Topology

**Figure 2:**
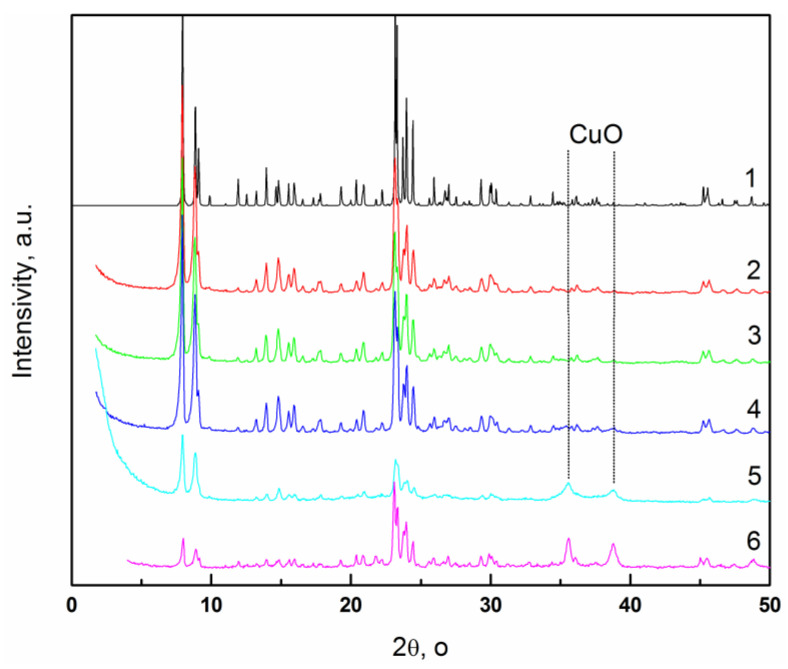
XRD patterns of Cu-MFI-SST samples with copper loadings 0 (1), 0.5 (2), 1 (3), 2 (4) and 16 wt.% (5). XRD of 16% Cu-ZSM-5-IMP presented for comparison (6).

The color of the copper-free and copper-containing ex-SST samples varies from light gray to dark gray, and becomes stronger when the copper content increases from 0.5 to 16 wt.%. The XRD patterns of the Cu-SST-series samples are typical of the crystalline silicalite with MFI structures (Figure 2). The intensities of the XRD reflections characterizing the MFI channel structure (at 2θ = 22–25°), which were observed for the Cu-MFI-SST samples with the low copper content (0.5–2 wt.%, Figure 2), are close to those of the copper-exchanged zeolites Cu-MFI-IEX and Cu-ZSM-5 as well as the impregnated Cu-zeolite [9]. The intensities of these reflections diminish when the copper content exceeds 10 wt.% in the Cu-MFI-SST and Cu-ZSM-5-IMP samples. This decrease is accompanied by a small shift of the reflections to larger angles. The changes observed in the diffraction patterns indicate that the MFI channel structure is disrupted and the channels are filled with CuO nanoclusters or finely dispersed CuO nanoparticles. Note that the decrease in the intensity of reflections at 2θ = 22–25° was more significant in 16%Cu-MFI-SST sample than in the impregnated sample 16%Cu-ZSM-5-IMP. Thus, Cu-SST sample is expected to contain more CuO nanoclusters and nanoparticles inside the zeolite channels compared to the impregnated catalyst. The 16%Cu-MFI-SST sample shows also the peaks belonging CuO crystallites (2θ = 35.4, 38.1°, Figure 2, curve 5). These characteristic peaks are very weak and broad in comparison with the XRD of the impregnated 16%Cu-ZSM-5-IMP sample (Figure 2, curve 6). This seems to evidence that in 16%Cu-MFI-SST copper oxide exists in small crystal nanoparticles with coherent-scattering region (CSR) around 20 nm. It can be argued that CuO nanoparticles are located on the external surface of zeolite crystallites because their CSR exceeds size of the zeolite channels (20 nm vrs. 0.54 × 0.56 nm). These data agree with those reported in [42].

So, the studied Cu-containing silicalites and alumosilicalites had MFI structure. Crystal CuO nanoparticles over zeolite crystallites formed in the Cu-MFI-SST, when the copper loading was as high as 10 wt.% and more. At low copper loading (up to 2 wt.% inclusive) in the Cu-MFI-SST samples, copper was probably inside the zeolite channels.

### 3.3. EPR and UV-Vis DR Study of Cu-Containing MFI-Silicalites and Zeolites with Low Copper Loading

#### 3.3.1. EPR Data

**Figure 3:**
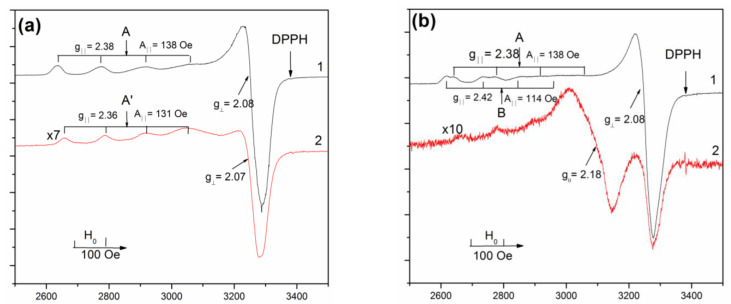
EPR spectra of Cu-MFI-SST samples recorded at −196 °C (1) and 25 °C (2). Copper loadings are 1 (**a**) and 16 wt.% (**b**).

**Figure 4:**
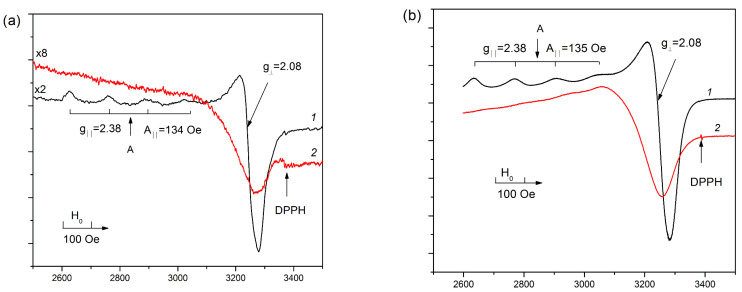
EPR spectra of the ion-exchanged Cu-containing MFI (**a**) and ZSM-5 (**b**) samples recorded at −196 °C (1) and 25 °C (2). Copper loading is 1 wt.%.

EPR spectra of the Cu-MFI-SST samples with different copper loadings prepared by the solid state transformation are presented in Figure 3. The values of g-factors and concentrations of spins observed by EPR are summarized in Table 3. EPR spectra of the isolated Cu^2+^ ions with axial anisotropy of g-tensor and the resolved hyperfine structure were observed at −196 °C (77 K) for the samples with the low copper concentrations (1 wt.% and less). Their EPR parameters g_‖_ = 2.38, A_‖_ = 138 G, and g_⊥_ = 2.08 are close to those for Cu-MFI-IEX (Figure 4a) and Cu-ZSM-5-IEX (Figure 4b) zeolites with the copper loading lower 2 wt.% supported by the ion-exchange (Table 3, Sample 5–9) or the incipient wetness impregnation (Table 3, Sample 10). The observed EPR spectral parameters are ascribed to the d_x_^2^_−y_^2^ ground state of Cu^2+^ ions stabilized in the octahedral oxygen coordination with a weak tetrahedral distortion (extension of the octahedron) [20,30,48,49,50,51], which is probably caused by nonequivalence of axial and equatorial ligands of the cation, e.g., H_2_O, O^2−^ and OH^−^ [49,50]. Herein, we denoted these isolated Cu^2+^ ions as Type A.

However, two specific features were observed in the EPR spectra of the Cu-SST-series samples with the low copper concentration (0.5%Cu-MFI-SST and 1%Cu-MFI-SST, Figure 3) in comparison with EPR spectra of the ion-exchanged Cu-MFI-IEX (Figure 4a) and Cu-ZSM-5-IEX samples (Figure 4b). First, the shape of the EPR spectra of the Cu-SST samples practically does not depend on the spectra registration temperature (−196 °C or 25 °C (298 K)). At both temperatures, the EPR spectra are characterized by an anisotropic signal with well-resolved four hyperfine components in the g_‖_ region with almost the same g-factors and line widths. The EPR parameters are g_‖_ = 2.38, A_‖_ = 138 G, g_⊥_ = 2.08 at −196 °C and g_‖_ = 2.36, A_‖_ = 130 G, g_⊥_ = 2.07 at 25 °C (Figure 3). These parameters indicate a slightly higher tetragonal distortion of the octahedron around the isolated Cu^2+^ ions at 25 °C compared to −196 °C, because at −196 °C a more stronger water coordination on Cu^2+^ ion results in a minor distortion of the octahedron. Note that the lines in the EPR spectra of the ion-exchanged Cu-MFI-IEX and Cu-ZSM-5-IEX samples are usually wide at 25 °C, so that the hyperfine structure is not well resolved (Figure 4, curve 2). The widening seems to result from mobility of water molecules in the coordination sphere of isolated Cu^2+^ ions [20,48].

The second feature of the EPR spectra of the Cu-SST-series samples with the low copper content is a significant (5–7-fold) difference in the intensities of the signals at 25 °C and −196 °C (Figure 3, curves 1 and 2), which exceeds that expected from the Curie law. Such substantial decrease in the EPR spectral intensity at 25 °C is probably due to the presence of clustered copper ions exhibiting antiferromagnetic interaction between the ions. It should be noted that paramagnetic Cu^2+^ ions in antiferromagnetic materials at temperatures exceeding the Neel point (T_N_), in particular at 25 °C vrs T_N_ ~ −60 °C (213 K) for bulk CuO [52], are characterized by a large dipole-dipole broadening of the EPR spectra in comparison with those recorded at −196 °C. This is due to difference in magnetic states (higher magnetic susceptibility) of oxide clusters at these temperatures.

Note that the amount of EPR visible paramagnetic Cu^2+^ ions in the Cu-SST-series (Table 3, samples 1–3) was lower compared with the ion-exchanged Cu-ZSM-5-IEX (Table 3, samples 7–10), but higher or comparable with the Cu-exchanged MFI samples (Table 3, samples 5, 6). At 2 wt.% of copper loading, the fraction of EPR visible Cu-ions decreased in order Cu-ZSM-5-IEX (80%) < Cu-ZSM-5-IMP (70%) < Cu-MFI-SST (30%) < Cu-MFI-IEX (25%). For the Cu-SST-series, the fraction of EPR-visible Cu^2+^ ions (relative to the total copper content in the sample) was the same (80–90%) at 0.5–1 wt.% of copper loading and decreased threefold with an increase in the copper content from 1 to 2 wt.%. These facts were caused by the different form of Cu^2+^ cations stabilization due to various anchoring sites in H-ZSM-5 zeolite and silicalites with MFI topology. For ion-exchanged samples, there are the isolated Cu^2+^ ions in the cation-exchange sites of H-ZSM-5 [20,22,29,30,51] and CuO-like nanoclusters entrapped within the channels and cavities of the MFI SiO_2_-framework [33,34,53]. The latter was proposed [34,53] to lose their antiferromagnetism, typical of large CuO particles, and become superparamagnetic due to their low nuclearity and the increased amount of surface Cu^2+^ ions, which are free to align with the magnetic field. By the XRD and EPR data, the main part of copper ions in the 2%Cu-MFI-SST samples is also in the clustered form and located inside the zeolite channels, but their behavior in magnetic field differ from the CuO-like nanoclusters in the ion-exchanged Cu-silicalite-1 samples. The assumption of the presence of copper oxide was proved by studying Cu-containing zeolites using UV-Vis DR spectroscopy and H_2_-TPR.

#### 3.3.2. UV-Vis DR Data

**Figure 5:**
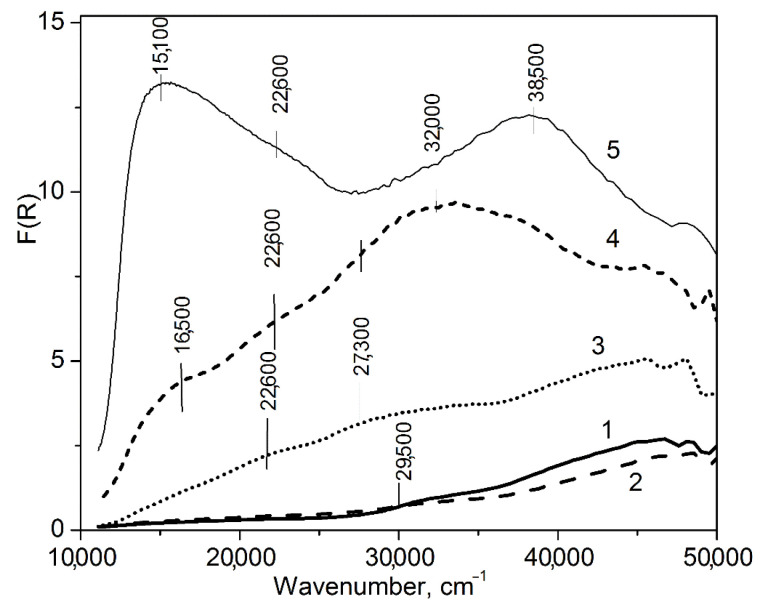
UV-Vis DR spectra of Cu-MFI-SST. The copper loadings are 0.5 (1), 1 (2), 2 (3) and 16 wt.% (4). For comparison, spectrum of CuO (5) is presented.

**Figure 6:**
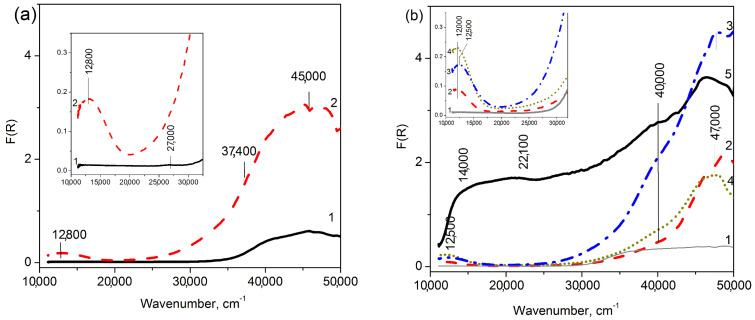
UV-Vis DR spectra of Cu-containing MFI (**a**) and ZSM-5 (**b**) prepared by the ion-exchange (2, 3) and incipient wetness impregnation (4, 5). The copper loadings are 0 (1), 2.0 (2, 4), 2.7 (3) and 16.0 (5).

UV-Vis DR spectra of the Cu-SST samples (Figure 5) are very different from the spectra of the ion-exchanged Cu-MFI (Figure 6a) and Cu-ZSM-5 (Figure 6b) samples with a low copper content. In the UV-Vis DR spectra of the Cu-SST-series samples, there were no distinct absorption bands. Only two broad shoulders with weak intensity at 13,300–14,100 and 29,000–33,000 cm^−1^ were distinguished. Their intensity was almost equal for the 0.5%Cu-MFI-SST and 1%Cu-MFI-SST samples, and it increased significantly for the 2%Cu-MFI-SST (Figure 5). The energy of the first and the second shoulders are typical of square-planar copper-oxide clusters characterized by d-d transition and ligand-to-metal charge transfer band (CTB O^2−^ → Cu^2+^) [20,21,54,55]. A substantial increase in the background absorption practically in the whole spectral range from 11,000 to 45,000 cm^−1^ was observed for 2%Cu-MFI-SST sample (Figure 5, curve 3). This absorption seems to prove the presence of finely dispersed CuO nanoclusters in the 2%Cu-MFI-SST sample, explaining the high proportion of EPR-silent Cu^2+^ ions (near 70%) in the sample; nevertheless, CuO clusters are very small, since they have not been registered by XRD.

For the ion-exchanged Cu-MFI and Cu-ZSM-5 samples (Figure 6), there are usually a well-defined absorption bands in the 12,000–13,000 cm^−1^ and 45,000–48,000 cm^−1^ regions [20,21], corresponding to the d-d transition of isolated Cu^2+^ ions (^2^T_2_g ← ^2^E_g_) in the weak-distorted octahedral field and their CTB O^2−^ → Cu^2+^, respectively [54]. At copper loading near 2 wt.%, their UV-Vis DR spectra did not contain the background absorption typical of bulk particles of CuO [56,57] and dispersed nanoclusters of CuO, as was observed for 2%Cu-MFI-SST. The broadening and shift towards higher wavenumbers of the d-d transition band, which are slightly noted in the spectrum of Cu-MFI-IEX (Figure 6a, curve 2), may indicate surface Cu^2+^ ions in CuO-like nanoclusters with low nuclearity [33,34]. In this case, the broadening of d-d transition band is caused by a tetragonal change in the octahedral symmetry around the Cu^2+^ ion due to a Jahn-Teller effect [20,55], leading to the splitting of levels of the excited T_2g_-term and the possibility of two transitions (instead of one) from the ground E_g_-state to the excited state [54,55]. The limiting case of an octahedron strong tetragonally distorted can be considered a square-planar environment of Cu^2+^ ions by oxygen-containing ligands. For square-planar symmetry of the crystal field, the typical energy of the d-d transition of Cu^2+^ ions is in the range of 13,300–14,500 cm^−1^ [20,33,54,55]. The absorption observed by us in spectra of Cu-MFI-SST samples with 0.5–1 wt.% of copper is included in this spectral region, but the absorption has the form of the wide shoulder, and not the band as in the spectra of the ion-exchanged Cu-silicalites with MFI zeotype (Figure 6a and [33]).

The absorption bands at 13,300–16,600 and 28,000–32,000 cm^−1^ were earlier observed in the UV-Vis DR spectra of some catalytic systems with low content of supported copper oxide, e.g., CuO/Al_2_O_3_ [56,57,58], CuO/ZrO_2_ [59,60] and CuO/CeO_2_ [58]. In these publications, the band at 14,300–16,600 cm^−1^ was attributed to the ^2^T_2g_ ← ^2^E_g_ transition of Cu^2+^ ions in the distorted octahedral site of the cubic lattice of CuO crystallites [56,60] or the CuO-like clusters located in surface defects of a support with the spinel-type structure [56] as well as on the surface of highly dispersed CuO nanoparticles having strong interaction with support [57,58]. The CTB O^2−^–Cu^2+^ near 40,000 cm^−1^ was assumed in [57,58,60] to be caused by the same Cu^2+^ ions that were characterized by the d-d transition energy of 14,300–15,400 cm^−1^; whereas, the absorption at 28,000–32,000 cm^−1^ was associated with Cu^2+^ of highly dispersed ion clusters [60] deposited on an oxide supports.

So, from the UV-Vis DR data, low copper-loading (up to 2 wt.%) silicalites with MFI zeotype prepared by the ion-exchange and solid state transformation are characterized by substantially different electronic states of Cu^2+^ ions. The ion-exchanged Cu-MFI is known to contain predominantly square-planar CuO-like nanoclusters with low nuclearity inside zeolite channels, and these Cu-species are detectable by wide absorption at 12,800–13,000 cm^−1^ with d-d transition energy. The ex-SST Cu-MFI samples comprise the clustering copper ions and the finely dispersed CuO-like nanoparticles, the spectral features of which are d-d transition of Cu^2+^ ions (13,300–14,100 cm^−1^)/CTB O^2−^–Cu^2+^ (at 29,000–33,000 cm^−1^) and the growth of the absorption background, respectively. The tendency of Cu^2+^ ions to the formation of CuO-like nanoparticles in the samples prepared by SST become stronger when the copper loading increases beyond 1 wt.%. Unlike both mentioned series of Cu-MFI samples, the ion-exchanged Cu-ZSM-5 samples have the isolated Cu^2+^ ions inside ZSM-5 channels that are formed due to the ion-exchange with protons of Si-OH-Al zeolite groups.

### 3.4. EPR and UV-Vis DR Study of Cu-Containing MFI-Silicalites and Zeolite with High Copper Loading

#### 3.4.1. EPR Data

The analysis of the EPR (Figure 3b) and UV-Vis DR (Figure 5, curve 4) spectra of the sample with 16 wt.% of copper introduced by the solid-state transformation gave unexpected results.

In addition to the Type A of the isolated Cu^2+^ ions, another type of isolated copper ions with parameters g_‖_ = 2.42, A_‖_ = 114 G, g_⊥_ = 2.08 (Type B, Figure 3b) is clearly observed in the EPR spectrum of the 16%Cu-MFI-SST sample at −196 °C. The parameters of its EPR spectrum correspond to weakening of tetragonal distortion of the octahedral symmetry compared to the Type A ions (g_‖_ = 2.38, A_‖_ = 138 G, g_⊥_ = 2.08), which are observed in the EPR spectra of the SST-series samples with the lower Cu loadings as well as of the ion-exchanged Cu-MFI (Figure 4a) and Cu-ZSM-5 (Figure 4b) samples. The EPR spectrum of the Type B ions disappears at 25 °C, with the simultaneous appearance of an almost symmetrical singlet with g_0_ = 2.18 and unresolved hyperfine structure. Note, the g_0_-factor observed in EPR spectrum of the 16%Cu-MFI-SST at 25 °C is close to the averaged value of g_‖_ and g_⊥_ of the isolated Cu^2+^ ion in the octahedral crystal field [61], realized as a result of the Jahn-Teller dynamic effect [61]. Thus, we believe that the symmetrical singlet with g_0_ = 2.18 corresponds to the Type B isolated Cu^2+^ ions observed at −196 °C, and both spectra are attributed to isolated Cu^2+^ ions with the octahedron coordination, for example, [Cu(H_2_O)_6_]^2+^ or [Cu(O)(H_2_O)_5_]^+^. The anisotropic EPR spectrum with quite close g-factors is well known for hydrated Cu-modified MOR [62] and BEA [63] zeolites.

Note that the fraction of isolated Cu^2+^ ions in the 16%Cu-MFI-SST sample is very low (less than 1%) even in comparison with the impregnated sample with the similar copper loading (the fraction of isolated Cu^2+^ ions in 16%Cu-ZSM-5-IMP is about 8%). The fact that only a minor fraction of copper ions are observed by EPR suggests that the majority of copper ions exists in the form of copper structures that are EPR-silent due to strong exchange interaction, e.g., copper oxide clusters, finely dispersed CuO nanoparticles or other groups of Cu^2+^-ions with the common oxygen-ligands. Besides, the EPR spectrum of 16%Cu-MFI-SST does not contain signals of weakly associated Cu^2+^-ions, which are characterized by an unstructured broad EPR signal (ΔH < 1000 G) with the slightly axial symmetry at −196–+25 °C [56,64]. These weakly associated Cu^2+^ ions are considered to clustered copper ions without common ligands and coupled via weak exchange interaction [56,64]. Such ions were observed for the Cu-containing zeolites with a variety of their framework topology: Cu-mordenite [25,30], Cu-Y [64], Cu-BEA [63] and for CuO/Al_2_O_3_ [56].

#### 3.4.2. UV-Vis DR Data

The main feature of the UV-Vis DR spectra of the 16%Cu-MFI-SST sample is the general intense absorption above 13,300 cm^−1^, on which several individual absorption shoulders at 16,500, 22,100 and 32,000 cm^−1^ (Figure 5, curve 4) can be distinguished. The 16%Cu-MFI-SST sample has the highest intensity of the absorption among the SST-series samples. The increased background absorption seems to be related with the semiconductor behavior of bulk CuO [56,57]. Indeed, according to the XRD data, the 16%Cu-MFI-SST sample contains CuO nanoparticles with the size about 20 nm (Figure 2, curve 5). Note that UV-Vis DR spectrum of 16%Cu-MFI-SST differs substantially from that of the impregnated sample with the similar copper loading, but containing CuO particles of 50 nm in size (16%Cu-ZSM-5-IMP, Figure 6b, curve 5). UV-Vis DR spectrum of the latter resembles that of bulk (unsupported) CuO (Figure 5, curve 5) [56,57]. There is an intense absorption in the regions from 14,000 cm^−1^ corresponding to the fundamental absorption edge (FAE) of CuO [57,59] and a broad shoulder at 38,500–40,000 cm^−1^.

The FAEs of CuO and Cu_2_O due to the band gap of bulk particles of CuO (*E*_g_ = 1.45–1.54 eV [65,66]) and Cu_2_O (*E*_g_ = 1.9–2.1 eV [66]) are usually visible in UV-Vis DR spectra at 11,600 and 15,300 cm^−1^, respectively. For CuO-supported materials, such as CuO/alumina and CuO/ZrO_2_, FAE is detected in the region of 15,300–16,500 cm^−1^ at copper loadings of 8–9 wt.% and higher [57,59]. To estimate the value of the optical band gap of CuO nanoparticles in the Cu-MFI-SST samples from their absorption spectra (Figure 5), the Tauc’s equation was used [67], and is written as follows:

$$\alpha =\frac{B{\left(hv-E_{g}\right)}^{n}}{hv_{}}$$

**Figure 7:**
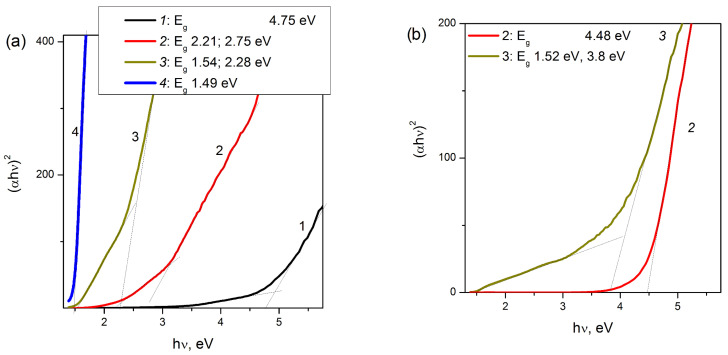
The (*αhv*)^2^ plot for determination of direct band gap for samples of Cu-SST-series (**a**), Cu-exchanged MFI (**b**, 2) and Cu-impregnated ZSM-5 (**b**, 3). The copper loadings are 1 (1), 2 (2) and 16 wt.% (3). Bulk CuO (4).

**Figure 8:**
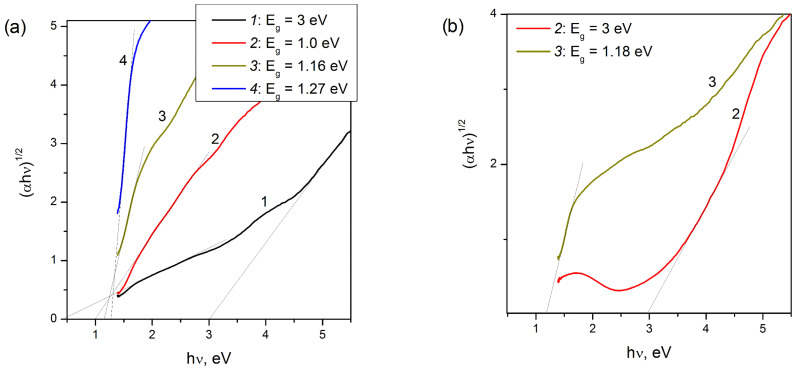
The (*αhv*)^1/2^ plot for determination of indirect band gap for samples of Cu-SST-series (**a**), Cu-exchanged MFI (**b**, 2) and Cu-impregnated ZSM-5 (**b**, 3). The copper loadings are 1 (1), 2 (2) and 16 wt.% (3). Bulk CuO (4).

The *hν* is the incident photon energy, *α* is the absorption coefficient, *B* is a materials dependent constant, *E_g_* is the optical band gap and *n* is a coefficient depending on type of electronic transition: ½ for direct allowed transition and 2 for indirect allowed transition. Figure 7 and Figure 8 display the plots of (*αhν*)*^1/n^* versus photon energy for Cu-MFI-SST samples. The value of *E_g_* was determined from the value of intercept of the straight line drawn to the first linear section of α-plot with the *x*-axis (that is, when *α* = 0). The values of direct (*n* = 1/2) and indirect (*n* = 2) band gap for CuO nanoparticles are shown next to the symbols on Figure 7 and Figure 8.

The indirect band gap of CuO nanoparticles in 2% Cu-MFI-SST and 16% Cu-MFI-SST was estimated to be 1.0 and 1.16 eV, respectively; it is red shifted ~0.27 eV and 0.11 eV as compared to value for bulk CuO (1.27 eV, Figure 8, curve 4). The increasing red shifts of indirect band gap with a decrease in the copper content (from 16 to 2 wt.%), and therefore with the assumed decreasing size of CuO nanoparticles, indicate that the defects responsible for the intra-gap states are primarily of surface defects. This trend is well complemented by the sample 16% Cu-ZSM-5-IMP with larger size of CuO particles (50 nm vrs. 20 nm) and E_g_ value (1.18 eV vrs. 1.16 eV) than that of 16% Cu-MFI-SST.

The estimated value of the indirect E_g_ for the 1% Cu-MFI-SST sample (Figure 8a, curve 1) was significantly lower than published in the literature (0.45 eV vrs. 1.2–1.54 eV [57,59,65]); thus, in the spectra of the low Cu-loaded SST-samples, the low-intensity absorption at 13,300–14,100 cm^−1^ is associated with the d-d transition of clustering Cu^2+^ ions in the CuO-like nanoclusters. Their d-d transition is consistent with CTB O^2−^–Cu^2+^ centered 29,000–33,000 cm^−1^. This is also confirmed by the fact that 80–90% of copper were EPR visible in them. Note that the shape of *α-*plots of 1% Cu-MFI-SST and 2% Cu-MFI-IEX (Figure 8b, curve 2) samples are very different, and for the latter there is no linear section in the photon energy range of 0–2.5 eV. Intensive absorption with energy of more than 4.4 eV is associated with the fundamental absorption edge of the zeolite [20,21], and it is clearly visible in the spectra of the Cu-zeolite samples with low copper loading (Figure 8).

The Cu-SST sample with 2 wt.% copper content showed higher value of the direct band gap (2.21 eV, Figure 7a, curve 2) compared to bulk value (1.49 eV, Figure 7a, curve 4, and 1.46 eV in [65]). The both high Cu-loaded samples (16 wt.%) had the direct band gap near 1.52–1.54 eV. The blue shift in the direct band energies is due to the quantum size effect [68].

**Figure 9:**
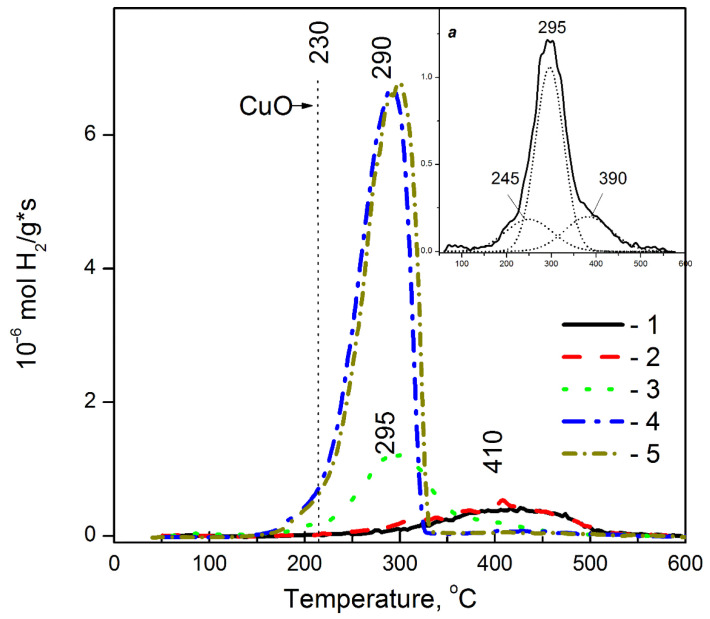
H_2_-TPR curves of Cu-containing MFI prepared by SST after pretreatment in O_2_ (1–4) or Ar (5) at 500 °C. The copper loadings are 0.5 (1), 1 (2), 2 (3) and 16 wt.% (4, 5). H_2_-TPR breakdown of 2%Cu-MFI-SST is presented on callout *a*.

So, the results obtained by EPR and UV-Vis DR spectroscopy suggest that the finely dispersed surface CuO nanoparticles interacting with the SiO_2_-framework are formed together with the square-planar CuO-like clusters in the sample with the high copper concentration (16 wt.%) synthesized by the solid-state transformation. Surface Cu^2+^ ions in the square-planar CuO-like clusters are registered by the EPR as the isolated Cu^2+^ ions, but their number is very small (no more than 1%). This conclusion agrees well with the data obtained by XRD (Figure 2, curve 5) and H_2_-TPR (Figure 9). The fine dispersion of CuO particles in the Cu-SST samples is either due to encapsulation of CuO species in the silica matrix or due to the uniform distribution of CuO on the silicalite matrix surface during SST synthesis of Cu-containing zeolites.

### 3.5. Redox Performance of Cu-Containing Zeolites

H_2_-TPR is a sensitive way to reveal additional information about the composition of active Cu structures, and to study their redox properties and thermal stability; moreover, the latter two characteristics often determine the catalytic behavior of Cu-containing zeolites in a number of redox reactions. H_2_-TPR profiles of the Cu-SST samples after treatment in oxygen and argon at 400 °C are shown in Figure 9. There is one wide asymmetric hydrogen consumption peak. One-step reduction is usually typical of bulk CuO [58]. The molar ration of the amount of hydrogen consumed during the TPR experiment to the copper content in the sample (H_2_/Cu) indicates that copper exists in the 2+ oxidation state (H_2_/Cu = 1.02–1.05) in all the samples. Taking into account the one-step copper reduction mechanism of the Cu-SST samples (Figure 9) assisted by the experimental molar ratio H_2_/Cu near 1, we believe that these samples contain CuO-like clusters and CuO nanoparticles. Both types of Cu-species are reduced by H_2_ according to overall reaction: CuO + H_2_ → Cu^0^ + H_2_O.

At the increase in the copper loading, the hydrogen consumption maximum shifts towards lower temperatures: from 400–420 °C for 0.5%Cu-MFI-SST to 280–300 °C for 16%Cu-MFI-SST (Figure 9). Still, it remains above the CuO reduction temperature both in the unsupported copper oxide (230–250 °C [58,69]) and the supported materials (210–260 °C [58,59,69,70,71,72]). Usually, the shift of hydrogen consumption by the supported CuO to lower temperatures is related to the increasing dispersity of CuO crystallites. This conclusion was made earlier for CuO/Al_2_O_3_ [58,71], CuO/ZrO_2_ [59] and CuO/SiO_2_ [72]. This effect becomes most pronounced when the surface CuO particles interact with a support which has high concentration of bulk oxygen vacancies (e.g., CuO/CeO_2_ [58,71]). According to the reported data, highly dispersed supported copper oxide species, which include weak magnetic associates, and small two- and three-dimensional clusters, are reduced at 210–220 °C [59,70,71], whereas large crystalline (bulk) CuO is reduced at 260 °C [59,70]. Meanwhile, the large crystallites of bulk Cu_2_O (330–350 °C [69,70]), the complex compounds, e.g., CuAl_2_O_4_ (around 500 °C [70,71]) and dioptase Cu_6_(Si_6_O_18_)·6H_2_O (around 430 °C [72]), and CuO encapsulated in the support matrix (at 530–600 °C [72]), undergo to reduction at elevated temperatures.

So, the difference in the reduction temperatures of the Cu-SST samples with various copper loadings demonstrates that the samples differ by the type of CuO-like species: clusters or nanoparticles, and their location in the silicalite. In particular, in the samples with 1 wt.% of copper or less (Figure 9, curves 1,2), which are characterized by the high-temperature hydrogen consumption peak (400–420 °C), copper seems to be present as Cu^2+^ ions in the square-planar CuO-like clusters encapsulated in the silicalite matrix. Apparently, their Cu^2+^ ions are EPR-active and give the signal of the isolated Cu^2+^ ions of Type A (Figure 3a).

In the Cu-SST samples with copper loadings 2 wt.% or more, surface CuO nanoparticles, which are reducible in hydrogen at 280–300 °C (Figure 9, curve 3), predominate (>70%). As the copper concentration grows, the size of the CuO nanoparticles increases while the fraction of Cu^2+^ ions and CuO-like clusters encapsulated in the silicalite matrix substantially decreases. The asymmetric shape of the hydrogen consumption peak typical of the 2%Cu-MFI-SST (Figure 9, callout *a*) and 16%Cu-MFI-SST (Figure 9, curve 4) samples reflects probably the presence of a small amount of highly dispersed surface copper oxide species, which are reduced at temperatures below 230 °C. The fraction of the latter is about 10% both for the 2%Cu-MFI-SST and for the 16%Cu-MFI-SST. In addition to these forms of copper, the 2%Cu-SST sample contains also poorly reducible Cu^2+^ ions (390 °C). Their fraction does not exceed 10% of total copper.

**Figure 10:**
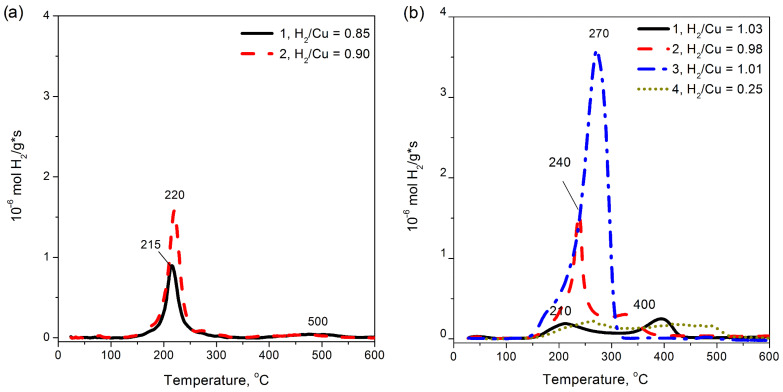
H_2_-TPR curves of Cu-containing MFI (a) and ZSM-5 (b) prepared by ion-exchange (1, 2, 3) and incipient wetness impregnation (4) after pretreatment in O_2_ (1, 2, 3) or Ar (4) at 500 °C. The samples are (**a**) 1.0%Cu-MFI-IEX (1), 2.0%Cu-MFI-IEX (2); (**b**) 1.0%Cu-ZSM-5-IEX (1), 2.8%Cu-ZSM-5-IEX (2 and 4), 16%Cu-ZSM-5-IMP (3).

The H_2_-TPR profile of the 16%Cu-MFI-SST sample (Figure 9, curve 4) pretreated in oxygen is practically identical to that of the impregnated 16%Cu-ZSM-5-IMP sample (Figure 10, curve 3). The maximum of reduction temperatures were 290 °C for the first and 270 °C for the latter. Given the XRD data, the enlargement of the CuO nanoparticle size is assumed to be the key reason to some improvement in the reducibility of the samples.

In contrast to the SST-series samples, the ion-exchanged 0.5–2%Cu-ZSM-5 samples contain predominantly isolated Cu^2+^ cations, which are characterized by two hydrogen consumption peaks in the H_2_-TPR experiments (Figure 10b, curve 1). The first peak at 210 °C can be attributed to the reduction in Cu^2+^ ions to Cu^+^; the second peak at 400 °C corresponds to the reduction in Cu^+^ to Cu metal [21,73,74,75]. Both reduction steps, Cu^2+^–Cu^+^–Cu, are characterized by molar H_2_/Cu = 0.5 [73,75]. A similar H_2_-TPR profile is typical of the 2%Cu-ZSM-5-IMP sample. An increase in the copper content in the ion-exchanged Cu-ZSM-5 samples up to 2.8 wt.% (Cu/Al > 0.5) by purposefully stabilizing square-planar CuO-like clusters inside ZSM-5 zeolite channels [75] leads to a sharp increase in the intensity of the low-temperature peak of hydrogen consumption. At the same time, the sharp shape of the peak and the temperature of its maximum (230–240 °C) just indicate the autocatalytic mechanism of two-electron reduction in CuO-like clusters [21,75].

On the other hand, the ion-exchanged Cu-MFI-IEX samples are characterized by a single-peak reduction profile, with maximum about 215–220 °C (Figure 10a). This sharp peak corresponds to a two-electron reduction in CuO-like clusters, as it was proved in [33]. The peak maximum is comparable to the temperature of the first reduction maximum observed in the H_2_-TPR profile of the ion-exchanged Cu-ZSM-5-IEX samples (Figure 10b), but significantly lower compared to the reduction temperatures of Cu-SST samples with low copper loading (Figure 9). When the copper content in the samples was 1 wt.%, maximum of their reduction temperatures raised in order and listed as follows: Cu-ZSM-5-IEX (210 °C) < Cu-MFI-IEX (215 °C) < Cu-MFI-SST (410 °C); at 2 wt.% of copper: 220 °C for Cu-MFI-IEX and 240 °C for Cu-ZSM-5-IEX vrs 295 °C for Cu-MFI-SST.

So, the key factor determining the difference in the reducibility of Cu-containing silicalite and zeolite is the copper electronic state. Both square-planar CuO-like clusters and CuO nanoparticles are reduced by hydrogen to metal in one-step, although the surface copper-oxide clusters are less resistant to hydrogen, while the finely dispersed CuO nanoparticles do so at higher temperatures, 230 vrs. 280–300 °C. The reduction in the isolated Cu^2+^ ions proceeds through an intermediate state of Cu^+^, while in some cases the transformation of Cu^2+^–Cu^+^ and Cu^+^–Cu can be strongly separated by temperatures (at least 150–200 °C). These ions are reduced in two stages at H_2_-TPR, but their reduction totally completed at temperature is lower than for the SST-samples.

The second feature distinguishing the SST-series samples is the similarity of the CuO-like species observed after treatment in oxygen and in argon (Figure 9, curves 4 and 5). This similarity indicates that the CuO-like species are stable with respect to thermal treatment in inert medium. In contrast to the Cu-SST-series samples, the concentration of CuO-like structures (such as square-plane CuO-like clusters) in the ion-exchanged (2.8%Cu-ZSM-5) and impregnated Cu-ZSM-5 samples with Cu/Al ≥ 0.5 [20,73,74] decreases after the treatment in argon at 400 °C (Figure 10, curve 4) in comparison with the samples pretreated in oxygen (Figure 10, curve 2).

### 3.6. Leaching Stability of Cu-Containing Zeolites Vrs Copper State

The differences in the dissolution kinetics of the samples prepared by the ion-exchange and solid-state transformation can be explained by variation of Cu-structures and their locations (surface, subsurface and inside channels) on the silicalites and zeolite.

The copper structures, which are localized on the surface and subsurface layers, are composed of oxide copper clusters and finely dispersed CuO nanoparticles. The surface copper-oxide clusters give d-d transition (13,300–15,300 cm^−1^) from the surface Cu^2+^-ions and CTB O^2−^–Cu^2+^ (shoulder at 28,000–32,000 cm^−1^). Finely dispersed CuO nanoparticles are EPR-silent. In their UV-Vis DR spectra, there is the increased general absorption with shoulders at 22,600 and 32,000 cm^−1^. Note, the surface CuO-like clusters are more characteristic for the ion-exchanged Cu-MFI samples, while the finely dispersed CuO nanoparticles are typical of the Cu-SST with copper content 2 wt.% and more. The chemical properties of the CuO-like clusters and the finely dispersed CuO nanoparticles are very similar. Both of them dissolve in the 1.2 M HCl solution during the differentiating dissolution due to the surface and subsurface localization of these structures in the silicalite matrix, as well as the property of CuO to interact with hydrochloric acid. The sufficiently high propensity of these structures to dissolve in acidic solutions prevents the use of catalysts based on them in reactions occurring in aqueous media (e.g., catalytic wet peroxide oxidation of Rhodamine 6G [13] and other dyes) due to the high leaching of the active component (achieving values as high as 80% [10]).

The isolated Cu^2+^ ions localized in the bulk of the SST-series samples and ion-exchanged samples are different. The SST-series samples contain two kinds of isolated Cu^2+^ ions. The first type of isolated Cu^2+^ ions is detected via the anisotropic EPR signal having similar parameters at −196 °C and 25 °C. The second is characterized by the isotropic signal with g_0_ = 2.18. Cu^2+^ ions detected in Cu-SST samples, probably, are encapsulated in silica matrix or strongly interact with it. As a result, they dissolve together with SiO_2_ matrix in HF and are subjected to the one-step reduction at 400–420 °C. Unlike Cu-SST, the ion-exchanged samples contain the isolated Cu^2+^ ions localized inside zeolite channels next to the zeolite ion-exchange positions Si(O^−^)Al. These ions are EPR-active; their anisotropic EPR signal is temperature-dependent. In the UV-Vis DR spectra, such copper ions are characterized by the absorption band at 12,500 cm^−1^. They are reduced in two stages at H_2_-TPR. So, these Cu^2+^ ions are similar to hexaaquacomplex [Cu(H_2_O)_6_]^2+^ and thus should be easily dissolved in the low-concentrated solutions of hydrochloric acid. However, these copper ions are releasing together with the elements of the zeolite matrix (Fe_0.005_Al_0.008_Si), most likely, due to some diffusion limitations. Apparently, the solvent access into the microporous zeolite channels and its removal from the channels with the dissolved Cu^2+^ ions is too slow because the differentiating dissolution is carried out in a dynamic mode.

## 4. Conclusions

The main electronic states of copper ions in the copper-containing silicalites with the MFI framework topology prepared by the solid-state transformation were studied. Several types of copper cations were determined in these samples: (1) two types of isolated Cu^2+^ ions (A and B) in the oxygen octahedral environment with different tetragonal (axial) distortion of the octahedron, which are low mobility due to their firmly attached to the CuO or SiO_2_ matrixes; (2) oxide clusters of the Cu^2+^ cations in the square-planar coordination of oxygen ligands, which are silica-decorated and are located in the silicalite subsurface; and (3) CuO nanoparticles dispersed on the silicalite surface and having the size down to 20 nm. The fractions of each state depend substantially on the copper loading. Its increase (2–16 wt.% Cu) tends to favor the formation of copper oxide clusters and copper oxide nanoparticles.

The electronic states of copper distinguished in the Cu-containing samples with the MFI topology prepared by the solid-state transformation, ion-exchange and incipient wetness impregnation were compared. It was shown that the first method largely favors clustering of copper ions, starting with the formation of the square-planar CuO-like clusters and ending with the formation of finely dispersed CuO nanoparticles in the Cu-MFI-SST samples with the high copper loading (about 16 wt.%). In the ion-exchanged Cu-MFI, one form of copper, such as surface square-planar CuO-like clusters, predominates. This is due to the absence of ion-exchange sites in the silicalite, and as a result Cu^2+^ ions are grafted on -SiOH groups of silicalite. In the Cu-ZSM-5 materials prepared by ion-exchange and incipient wetness impregnation, copper ions are stabilized primarily as isolated ions when Cu loading does not exceed 2 wt.%.

Although the isolated Cu^2+^ ions, which are stabilized in the solid-state transformation, the ion-exchanged and impregnated samples everywhere, have axial-distorted octahedral coordination, they differ by the ligand nature and distortion degree. The main difference of isolated ions stabilized in the SST samples from those in the ion-exchanged and impregnated samples is their rigid attachment into the crystalline lattice of silicalite, most likely, in sites different from the zeolite ion-exchange sites. Such firm attachment of the isolated Cu^2+^ ions results in the decrease in the redox properties and activity with respect to different solvents in the case of samples with the 0.5–1 wt.% of copper. The CuO-like clusters located on the surface and in the subsurface layers of the samples prepared by the solid-state transformation are reduced with more difficulty than those in the ion-exchanged and impregnated samples, though their activity to dissolution in HCl solutions are similar to the ion-exchanged Cu-MFI (silicalite). It caused by decoration with the silica matrix. The redox activity of the finely dispersed CuO nanoparticles located on the surface of the samples prepared by the solid-state transformation and impregnation and their activity to dissolution in HCl solutions are close even though their sizes are different (20 vrs. 50 nm). Cu^2+^ ions of Cu-ZSM-5 located inside zeolite channels are the most difficult to leaching, probably, due to some diffusion restrictions.

## Figures and Tables

**Table 1:** The chemical compositions of Cu-containing zeolites prepared by different methods.

	Sample	Method of Cu Introduction	Chemical Composition, wt.%				
			Cu	Na	Fe	Al	Si
1.	0.5%Cu-MFI-SST	SST	0.49	2.94	0.08	0.19	44.8
2.	1.0%Cu-MFI-SST	SST	1.18	2.73	0.13	0.21	45.1
3.	2.0%Cu-MFI-SST	SST	2.72	1.92	0.07	0.14	43.7
4.	16.0%Cu-MFI-SST	SST	16.1	0.14	0.06	0.14	37.3
5.	1.0%Cu-MFI-IEX	IEX	0.95	0.05	0.07	0.30	43.4
6.	2.0%Cu-MFI-IEX	IEX	2.03	0.06	0.09	0.27	42.7
7.	0.5%Cu-ZSM-5-IEX	IEX	0.47	0.06	0.65	1.43	42.9
8.	1.0%Cu-ZSM-5-IEX	IEX	1.05	0.05	0.65	1.40	42.9
9.	2.0%Cu-ZSM-5-IEX	IEX	1.97	0.05	0.65	1.41	42.9
10.	2.0%Cu-ZSM-5-IMP	IMP	2.01	0.06	0.65	1.43	42.8
11.	16.0%Cu-ZSM-5-IMP	IMP	16.0	0.05	0.65	1.43	42.8

**Table 2:** Results of the chemical differentiating dissolution of Cu-containing zeolites.

N	Sample	Fragmentary Chemical Composition of Dissolved Species and Phases	Phase Contents, wt.%	Hypothesized Structural Compound
1	0.5%Cu-MFI-SST	Cu(II), soluble in H_2_O	<0.05 (3% Cu)	CuO on external surface of silicalite
		Cu(II), soluble in 1.2 M HCl, part of them dissolved with SiO_2_	2 (67% Cu)	CuO with strong interaction with external surface of silicalite
		Al_0.001_Si_1_ containing Cu(II) with Cu_0.0015_Si_1_ and Cu_0.30_Al_1_, it is soluble in 3.6 M HF	98 (30% Cu)	Cu^2+^ ions, in cation-exchange sites of MFI, bulk of Al-silicalite
2	2.0%Cu-MFI-SST	Cu(II), soluble in H_2_O	0.14 (7% Cu)	CuO on external surface of silicalite
		Cu(II), soluble in 1.2 M HCl, part of them dissolved with Si-matrix	1.8 (91% Cu)	CuO with strong interaction with external surface of silicalite
		Al_0.0008_Si_1_ containing Cu(II) with Cu_0.0001_Si_1_ and Cu_0.01_Al_1_, it is total soluble in 3.6 M HF	97 (2% Cu)	Cu^2+^ ions, in cation-exchange sites of MFI, bulk of Al-silicalite
		Al soluble in 3.6 M HF	0.2	(AlO)x-like, extralattice Al^3+^ ions
3	16%Cu-MFI-SST	Cu(II), soluble in H_2_O	0.8 (5.4% Cu)	CuO on external surface of silicalite
		Cu(II), soluble in 1.2 M HCl, part of them dissolved with SiO_2_	14.9 (93% Cu)	CuO with strong interaction with external surface of silicalite
		Al_0.001_Si_1_ containing Cu(II) with Cu_0.0001_Si_1_ and Cu_0.013_Al_1_, it is total soluble in 3.6 M HF	84 (2.7% Cu)	Cu^2+^ ions, in cation-exchange sites of MFI, bulk of Al-silicalite
4	2.0%Cu-MFI-IEX	Cu(II), soluble in H_2_O	1.3 (65% Cu)	CuO on external zeolite surface
		SiO_2_ containing Cu(II), both are soluble in 1.2 M HCl	9.0 (33% Cu)	CuO (decorated by SiO_2_) in near-surface layers of zeolite crystal
		Al_0.003_Si_1_ containing Cu(II) with Cu_0.04_Al_1_, both are soluble in 3.6 M HF	91 (2% Cu)	Cu^2+^ ions, in cation-exchange sites, bulk of silicalite
		Al soluble in 3.6 M HF	0.2	(AlO)x-like, extralattice Al^3+^ ions
5	2.0%Cu-ZSM-5-IEX	Cu(II), soluble in 1.2 M HCl	0.5 (2.7% Cu)	CuO-like clusters
		Al_0.008_Fe_0.003_Si_1_ containing Cu(II) with Cu_0.0004_Si_1_, both are soluble in 3.6 M HF	98 (97.3%Cu)	Cu^2+^ ions, in cation-exchange sites, bulk of zeolite
		Al^3+^, soluble in 1.2 M HCl and 3.6 M HF	0.1%	(AlO)x-like, extralattice Al^3+^ ions

**Table 3:** The main electronic state of Cu-ions in Cu-containing zeolites prepared by different methods and their UV-Vis DR and EPR characteristics.

	Sample		Ab. Band in UV-Vis DR, cm^−1^		EPR Parameters at −196 °C				
			d-d Cu^2+^_isol_	CTB L → M	g_||_	A_‖_	G_⊥_	Spin/g	%Cu ^a^
1.	0.5%Cu-MFI-SST	Cu^2+^, D_4h_	14,100 ^b^	32,000 ^c^	2.38	138	2.08	3.8 × 10^19^	80
2.	1.0%Cu-MFI-SST	Cu^2+^, D_4h_	14,100 ^b^	32,000 ^c^	2.38	138	2.08	8.4 × 10^19^	90
3.	2.0%Cu-MFI-SST	Cu^2+^, D_4h_ CuO-like	- 16,500 ^b^	32,000 ^c^ 22,100 ^d^	2.38	138	2.08	5.6 × 10^19^	30
4.	16.0%Cu-MFI-SST	Cu^2+^, D_4h_ CuO-like	- 16,500 ^b^	32,000 ^c^ 22,100 ^d^	2.38 2.42	138 114	2.08	1.4 × 10^19^	1
5.	1.0%Cu-MFI-IEX	Cu^2+^, O_h_	12,800	32,000 ^c^ 45,000	2.38	134	2.08	5.7 × 10^19^	45
6.	2.0%Cu-MFI-IEX	Cu^2+^, O_h_	13,000	32,000 ^c^ 45,000	2.38	134	2.08	3.1 × 10^19^	25
7.	0.5%Cu-ZSM-5-IEX	Cu^2+^, O_h_	12,000	47,500	2.38	135	2.07	6.0 × 10^19^	95
8.	1.0%Cu-ZSM-5-IEX	Cu^2+^, O_h_	12,000	47,500	2.38	135	2.07	1.2 × 10^20^	95
9.	2.0%Cu-ZSM-5-IEX	Cu^2+^, O_h_	12,700	47,500	2.38	135	2.07	9.6 × 10^20^	80
10.	2.0%Cu-ZSM-5-IMP	Cu^2+^, O_h_	12,500	-	2.38	138	2.08	1.4 × 10^20^	70
11.	16.0%Cu-ZSM-5-IMP	Cu^2+^, O_h_	13,300	-	2.38	138	2.08	1.3 × 10^20^	8

^a^—fraction of the copper observed by EPR to the copper concentration determined by IPC-AES (%); ^b–d^—observed as shoulders over general absorption (FAEs of zeolite and CuO nanoparticles).

## Data Availability

Data are contained within the article.
